# Genetic, hormonal and metabolic aspects of PCOS: an update

**DOI:** 10.1186/s12958-016-0173-x

**Published:** 2016-07-16

**Authors:** V. De Leo, M. C. Musacchio, V. Cappelli, M. G. Massaro, G. Morgante, F. Petraglia

**Affiliations:** Department Molecular Medicine and Development, University of Siena, Policlinico Le Scotte, Viale Bracci, 53100 Siena, Italy

**Keywords:** PCOS, Genetic, Insulin-resistance, Hyperandrogenism, Infertility, Metformin, Oral contraceptives, Myo-inositol

## Abstract

Polycystic ovary syndrome (PCOS) is a complex endocrine disorder affecting 5–10 % of women of reproductive age. It generally manifests with oligo/anovulatory cycles, hirsutism and polycystic ovaries, together with a considerable prevalence of insulin resistance. Although the aetiology of the syndrome is not completely understood yet, PCOS is considered a multifactorial disorder with various genetic, endocrine and environmental abnormalities. Moreover, PCOS patients have a higher risk of metabolic and cardiovascular diseases and their related morbidity, if compared to the general population.

## Background

### Definition and diagnostic criteria

Polycystic ovary syndrome (PCOS) is the most common endocrine disorder in women and major cause of anovulatory infertility. PCOS patients can present a wide range of signs and symptoms, which make difficult the precise grading of the condition. Diagnosis of PCOS is currently based on the criteria of the ESRHE/ASRM Rotterdam consensus meeting in 2003 [1], which broadened the previous NIH classification of 1990 [2]. It based on at least two of the following features: oligo-anovulation, hyperandrogenism and polycystic ovaries by ultrasound [1]. In 2006, the Androgen Excess Society (AES) set up a committee of experts to review all the data published on PCOS for the purpose of simplifying diagnosis [3]. The AES criteria require clinical and/or biochemical hyperandrogenism simultaneously with oligo/anovulation and ultrasonographic evidence of polycystic ovaries.

Although the aetiology of PCOS is not completely understood yet, PCOS is considered a multifactorial disorder with various genetic, metabolic, endocrine and environmental abnormalities [4]

There is increasing evidence suggesting that PCOS affects the whole life of a woman, can begin *in utero* in genetically predisposed subjects, it manifests clinically at puberty, continues during the reproductive years. It can also expose patients to increased risk of cardiovascular disease, hypertension, diabetes and other metabolic complications, especially after menopause [4]. During the fertile period it may cause anovulatory infertility and could be associated with increased prevalence of gestational complications, such as miscarriage, gestational diabetes and preeclampsia [5]. Early diagnosis is therefore crucial by enabling close follow-up and in an attempt to reduce the risk of such complications.

It is now widely recognised that insulin resistance, manifesting above all in obese or overweight women, but often also in lean PCOS women, is one of the key to this complex disorder. It determines hyperandrogenism by acting synergically with luteinising hormone (LH) on ovarian steroidogenic enzymes and on sex hormone-binding globulin (SHBG) production by the liver [5].

Diagnostic workup includes hormonal evaluation of androgen levels, clinical evaluation of hirsutism trough Ferriman-Gallwey score and ultrasonographic examination of the number of antral follicles and ovarian volume. Insuline resistance should be evaluated by HOMA INDEX (product of fasting plasma insulin [mU/L] and glucose [mmol/L] concentrations divided by 22.5). Future diagnostic approaches could be ultrasonographic 3D evaluation of follicles and is under discussion the role of anti-mullerian hormone (AMH) [6, 7].

### Etio-pathogenesis and pathophysiology: role of genetic, environmental and endocrine factors

Genetic and endocrine factors, together with environmental influences. In the research of etiopathogenesis of the syndrome and in the subsequent pathophysiological expression play a role genetic and endocrine as well as environmental factors. The most interesting hypothesis was proposed by Franks et al. [4], who defined PCOS as a genetically determined ovarian pathology characterised by over-production of androgens and manifesting heterogeneously according to the interaction of this genetic “predisposition” with other genetic and environmental factors. This hypothesis is persistent by the finding of polycystic ovaries in pre-pubertal girls [4, 8]. Studies in rhesus monkeys have demonstrated that exposure of foetuses to high levels of androgens during intrauterine life determines the onset of clinical manifestations of PCOS during adolescence. Studies in sheep have shown that an excessive androgen exposure during foetal life influences early ovarian follicular activity and it may explain the typical altered folliculogenesis shown in PCOS [4, 8].

The aforementioned observations may suggest that exposure of the foetal hypothalamus-pituitary-ovarian axis to androgen excess may trigger a series of events that could determine PCOS onset of at puberty.

The source of intra-uterine androgens excess is unlikely to be maternal, since the foetus is protected by placental aromatase activity and by high maternal SHBG concentrations.

The expression of aromatase in the placenta of PCOS women may be diminished [9] and this could potentially be unable to prevent foetal testosterone (T) excess in PCOS pregnancies [10]. It has been seen that the prevalence of decreased aromatase required to carry out T excess in female fetuses was reported to be extremely rare [11]. On the other hand, recent studies on hypertensive preeclamptic pregnancies have demonstrated a significant reduction in placental ability to synthesize oestrogens, indicating a gestational impairment of T aromatization that is more common than was previously considered [12, 13].

The source of androgens excess is more likely to be the foetal ovary, which is normally quiescent, but it could produce an excess of androgens in response to maternal hCG in subjects genetically predisposed to PCOS.

In newborn daughters of PCOS women, elevated T levels have been observed in the umbilical venous blood [14, 15]. This finding was not confirmed in other studies that demonstrated instead a reduced umbilical cord blood androstenedione levels [9, 16]. Hichey et al., showed no increase in T levels in umbilical cord blood of adolescent girls diagnosed with PCOS [17]. Taken the ovary as a key foetal site for gestational T excess, during critical mid-gestational age for target organ differentiation [9], studies at the time of birth, are likely to be too late to detect any remaining hormonal differences [18, 19]. The mid-gestational T excess in human female foetuses can be accompanied by gestational hyperglycaemia and foetal hyperinsulinemia. Interestingly, elevated mid-gestation maternal T levels predict high AMH levels in adolescent daughters [20]. Since elevated AMH represents a characteristic of adolescents and women with PCOS [21] and daughters of PCOS women [22, 23], such associations might suggest a cross-generational relationship between the degree of maternal hyperandrogenism and the development of PCOS in their daughters.

**Fig. 1:**
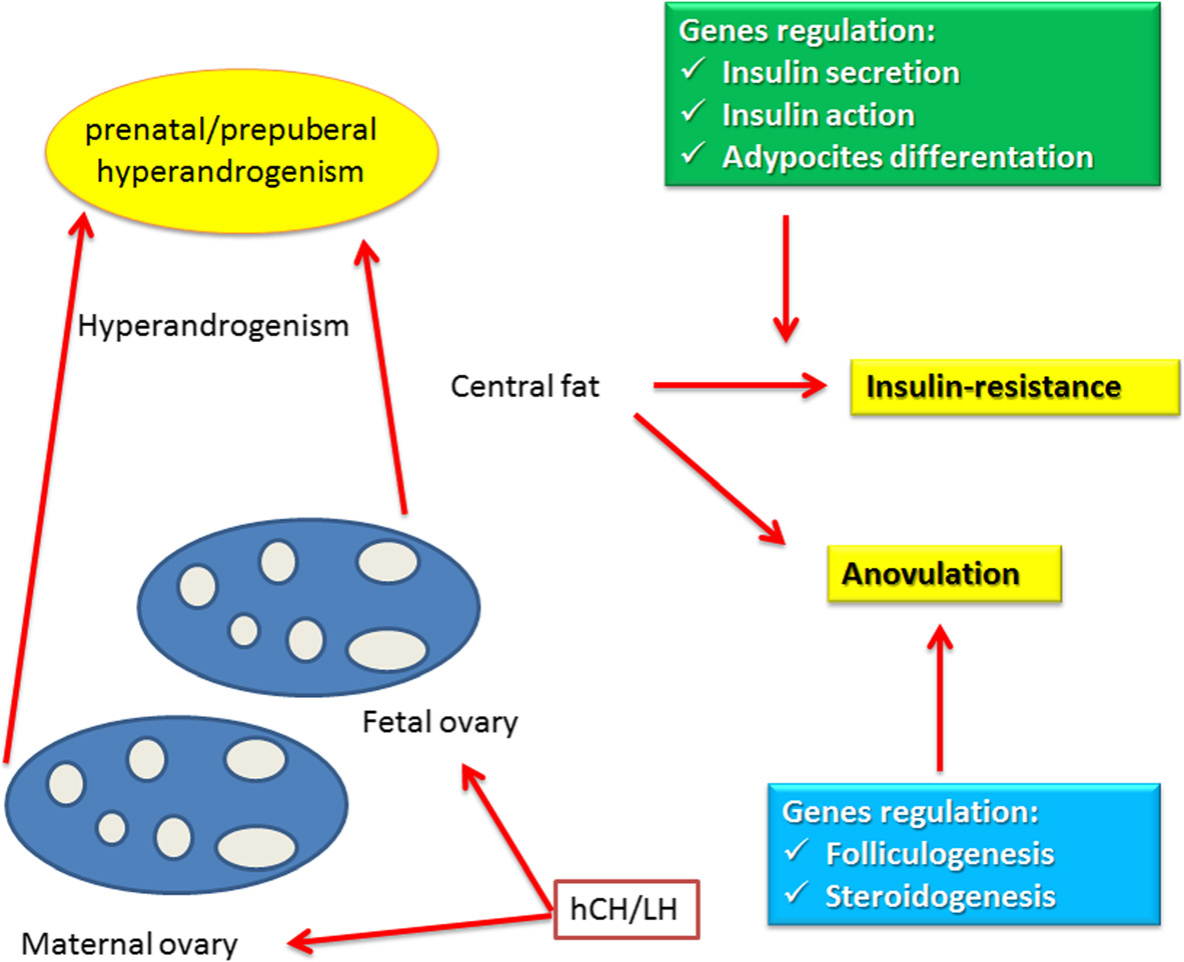
Theory of prenatal origin of PCOS and its development at puberty

Complete manifestation of the syndrome occurs at adolescence, when the hypothalamus-pituitary-ovarian access is activated. At this time, metabolic changes leading to modifications in the distribution of body fat also occur. In particular, at puberty there is a physiological increase in insulin levels, determining on one hand a reduction in SHBG levels with amplification of the effects of circulating androgens, and on the other hand, direct stimulation of ovarian steroidogenesis [24]. In women with PCOS, the physiologic hyperinsulinemia of adolescence may be a triggering factor for the development of hyperandrogenism and anovulation. Girls predisposed to insulin-resistance and overweight are even more at risk of developing early adrenarche and subsequent PCOS at adolescence [24] (Fig. 1).

Daughters of women with PCOS evaluated during early childhood (age 4–8 years) and early puberty (age 9–13 years) have exaggerated adrenarche compared with daughters of non-PCOS women of similar pubertal stage and body mass index (BMI) [25]. This is consistent with a role of obesity-related insulin- resistance in causing hyperandrogenemia in these girls through an effect of insulin on adrenal and ovarian steroidogenesis [26], manifesting as early adrenarche [27] and subsequent PCOS [28]. Such hyperandrogenemia appears to modulate gonadotropin levels, as has been demonstrated in obese peri-pubertal girls who were found to have increased LH frequency but low LH amplitude, and diminished overnight LH pulse amplitude compared with normal-weight girls [29]. These changes may reflect initial effect of obesity on LH pulses [30]. Subsequently, hyperandrogenemia reduces the inhibition of GnRH pulse frequency by progesterone, causing rapid LH pulse secretion and further increase in ovarian androgen production [30–32].

### Epigenetics and PCOS

Since the development of other diseases in adulthood, induced by nutritional or environmental factors in utero, usually involves an epigenetic mechanism, it seems likely that the same mechanism may also occur in PCOS. According to this hypothesis, exposure to hyperandrogenism *in utero* may lead to epigenetic reprogramming anomalies in foetal reproductive tissue, which is translated into the PCOS phenotype in adulthood. Moreover, if such epigenetic alterations persist in the germ cell line, transgenerational transmission of the PCOS phenotype is promoted. Clearly other genetic factors (i.e. linked to insulin resistance) and post-natal environmental factors (i.e. diet) may contribute to the development of PCOS phenotype, possibly in association with epigenetic anomalies.

In particular, epidemiologic and clinical studies conducted largely in adult human populations suggest a link between foetal growth restriction, and subsequent risk of type 2 Diabetes Mellitus (DM) and cardiovascular disease [33, 34]. The increased risk for these metabolic diseases has been linked to elevated insulin resistance in young individuals exposed to an adverse in utero environment and born small for gestational age (SGA) [35, 36]. These studies support an overall relationship between foetal growth restriction and increased adiposity and insulin resistance starting early in the childhood period. In addition to the in utero environmental factors, genetic polymorphisms may modulate insulin resistance parameters in SGA individuals. This may partially explain the variable degree of insulin resistance in subjects exposed to an adverse in utero environment [37]. At the other extreme, over-nutrition of theses foetuses appears to have long-term effects on obesity, insulin resistance, and predisposition to disorders of glycemic regulation. Offspring of mothers with diabetes during pregnancy have a higher frequency of childhood obesity and earlier onset of impaired glucose tolerance [38, 39] and type 2 DM [40]. Given the effect of insulin on modulating ovarian [41] and adrenal [42] steroidogenesis, a role of intrauterine adverse events which lead to insulin resistance and/or hyperinsulinemia may predispose adolescents to PCOS. Overall, these studies indicate that at least some metabolic components of the PCOS phenotype are programmed in utero, particularly the tendency for higher fat mass, visceral adiposity, and insulin resistance.

If further research verifies this hypothesis, new prospects for preventive treatment during the critical prenatal period will be mandatory.

### Genetic factors

Increasing evidences over many years point to familial aggregation of women with PCOS, hyperandrogenism and metabolic alterations [43]. The model of inheritance of PCOS has not yet been defined. Some researchers have postulated autosomal dominant transmission linked to a single genetic defect, but most authors define PCOS as a polygenic pathology. It is also possible that a particular gene in a given family may have a predominant effect, influencing the phenotypic manifestations of the syndrome. The main candidate genes are those encoding for factors involved in the synthesis, transport, regulation and effects of androgens. Other candidate genes are those encoding for factors involved in insulin metabolism, such as insulin receptors, signalling cascade proteins responsible for binding of insulin to its receptor, IGF system, other growth factors and the gene encoding for Calpain-10 enzyme, responsible for insulin secretion and action [43].

An association has also been found between “pro-inflammatory” genotypes and PCOS, linked to polymorphism of genes coding for TNF-alfa, IL-6 and IL-6 receptor [43]. Finally, recent evidence of altered early gonadotropin-independent folliculogenesis in women with PCOS suggests that genes involved in folliculogenesis may also be candidates in the etiopathogenesis of this syndrome [1].

However, only a few PCOS susceptibility genes have been repeatedly identified in studies of women with Chinese or European ancestry: allelic variants of fibrillin-3 (FBN3) [44–47], and variants of LH receptor (LHR) [44, 48, 49]. FBN3 encodes for an extracellular matrix protein that regulates transforming growth factor (TGF) signaling. Its PCOS associated allelic variant, A8, manifests a metabolically distinct phenotype, including insulin resistance [50]. FBN3 expression, is limited to early to mid-gestation in many organs and tissues, including the ovary [51, 52]. Such a gestational stage includes a period of foetal developmental at which T exposure induces altered DNA methylation of TGF-beta–regulating genes and subsequent PCOS-like traits [53]. Due to the degree and type of fibrillin expression contributes to differences in elasticity of cell extracellular matrix interactions and storage of TGF-beta, fibrillin may provide gestationally relevant [51] tissue-specific bases for cell mediated engagement of extracellular matrix–stored TGF-beta in proliferation, differentiation, and apoptosis [54, 55]. In the ovary, variants of LHR may diminish or enhance pituitary LH stimulation of ovarian theca and stroma cell T production, ovarian follicle development, LH surge–induced ovulation, and corpus luteum function [56], while in adipocytes, LHR variants may alter LH stimulation of adipogenesis [57]. Variants in these multi-organ system genes could contribute to genetically determination of PCOS phenotypes for reproductive and metabolic pathophysiology.

### Environmental factors

Although the prevalence of PCOS is similar in all countries, ethnic factors influence the phenotypic manifestations of the syndrome. The prevalence of PCOS among Caucasian women, varies from 4.7 % in Alabama, to 6.5 % in Spain and 6.8 % in Greece [58]. In the United Kingdom, PCOS and type II diabetes are more frequent in women of Asian origin [58]. These observations suggest the existence of different environmental factors, such as diet, physical activity and life-style in general.

The increasing effects of metabolic disorders in economically developed countries has led authors to suggest that the pathogenic mechanisms of these disorders are associated with evolutionary advantages in terms of survival [58]. On one hand, insulin resistance increases the availability of glucose for brain metabolism, while on the other, it increases blood pressure by mechanisms such as fluid retention and increase in the sympathetic tone. It also induces modifications in clotting factors (hypercoagulation) and a propensity for obesity characterised by a proinflammatory condition with increased secretion of cytokines and inflammatory factors. All the aforementioned alterations make the subject more resistant and favours survival when faced with stressors such as reduced availability of food, wounds and epidemics. The relative infertility of these women increases the interval between pregnancies and reduces the number of children, favouring survival of mothers and children. In the absence of stressors, as in the case of developed countries, these pathogenic mechanisms predispose to cardiovascular disease and atherosclerosis.

### Endocrine factors

**Fig. 2:**
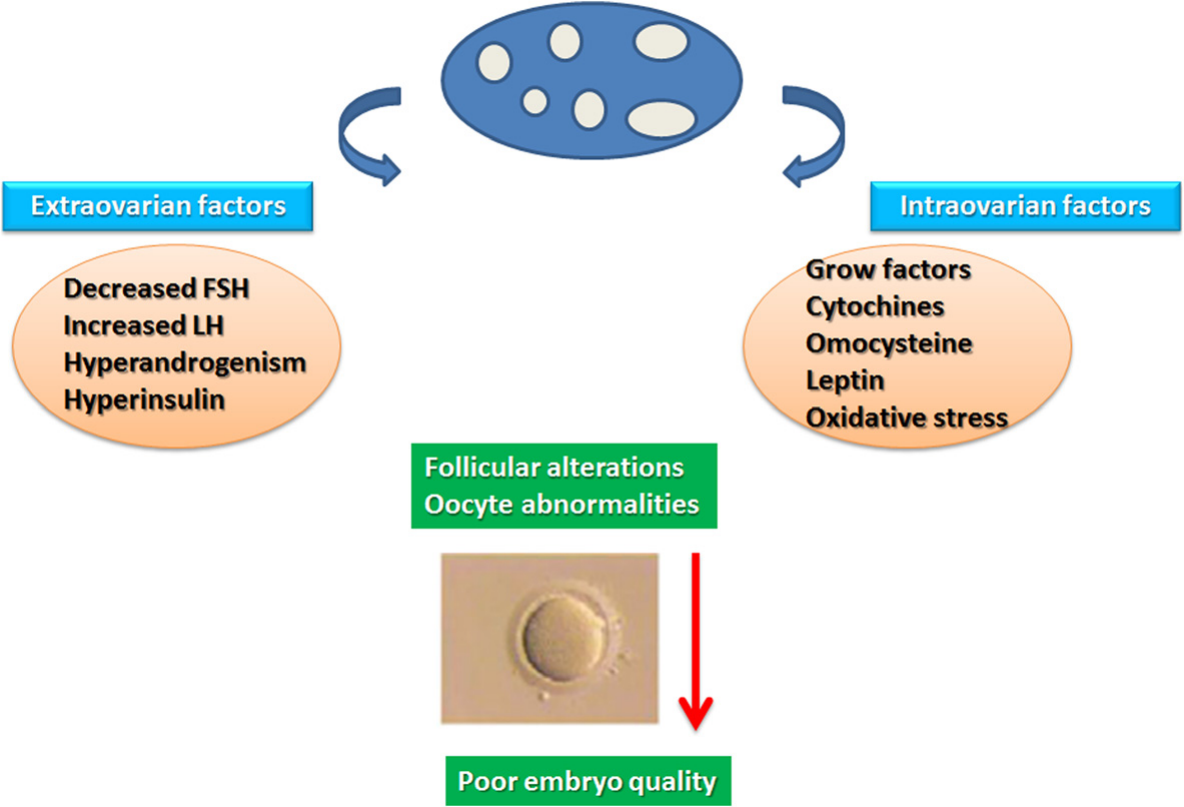
Alteration of extra- and intra-ovarian factors may compromise follicular development and oocyte development in PCOS

Ovarian folliculogenesis is regulated by a delicate equilibrium between extra- and intra-ovarian factors. Disturbance of this equilibrium may alter and compromise follicular development and the formation of mature oocytes, leading to infertility (Fig. 2).

### Extraovarian factors

Extraovarian factors include a series of endocrine, paracrine and metabolic alterations, which by causing abnormalities in the follicular microenvironment, may alter folliculogenesis and oocyte development. These alterations include FSH deficit, hypersecretion of LH, hyperandrogenemia of ovarian or adrenal origin and hyperinsulinemia with insulin resistance [59]. Folliculogenesis and oogenesis also depend on intraovarian factors, especially follicular fluid factors (FFFs) [59] that are directly correlated with their levels in plasma. Recent studies suggest that FFFs implicated in folliculogenesis of polycystic ovaries belong to the family of growth factors including cytokines and inhibins [59].

Vitamin D is an essential regulator of bone and mineral homeostasis. Recent studies have demonstrated hypovitaminosis D is associated with an increased likelihood of developing metabolic disorders. [60]. Vit.D deficiency has also been demonstrated in patients with POCS [61]. Obese patients with PCOS have been shown to have lower serum levels of 25-OH-D than non obese women with PCOS and vitamin D deficiency has been suggested to have a role in the development of insulin resistance (IR) and impaired glucose tolerance in such patients [62]

### Altered secretion of GnRH and gonadotropins

**Fig. 3:**
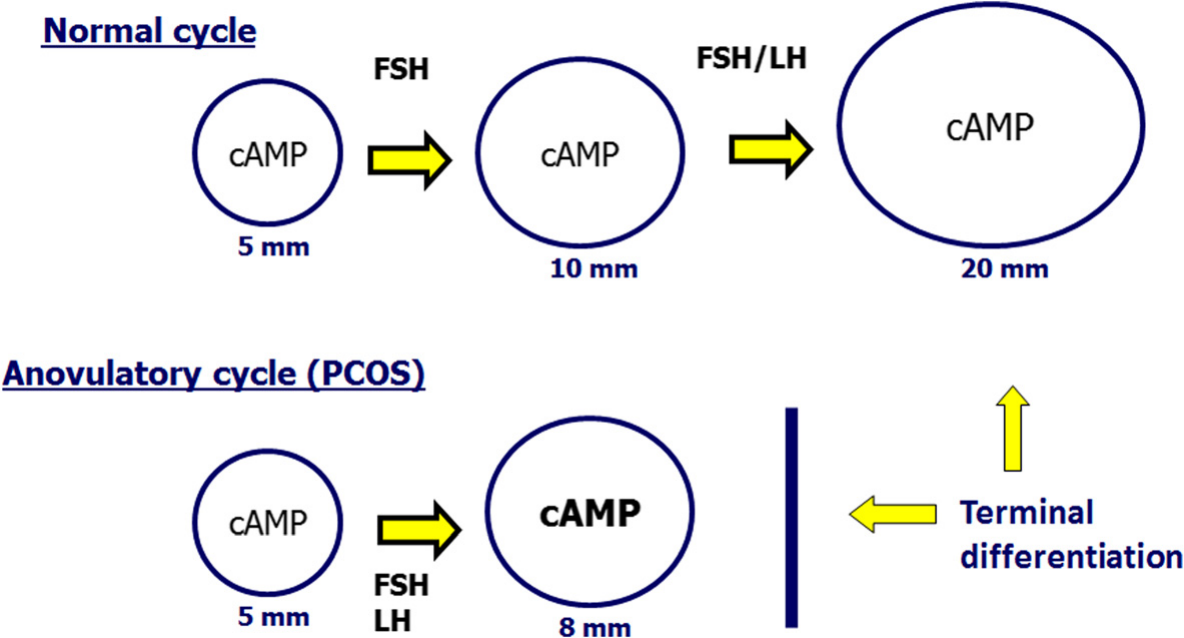
In a normal cycle, only the dominant follicle responds to LH when it reaches 10 mm diameter. In PCOS the response to LH occurs inappropriately in smaller follicles and many antral follicles reach terminal differentiation before time, producing a greater quantity of steroids and inhibin B that exert negative feedback on FSH production

Although the etiopathogenesis of PCOS is still controversial, series of hypotheses have been proposed in the recent decades. A high percentage (55–75 %) of women with PCOS have an elevated LH/FSH ratio presumably due to high levels of LH rather than reduced production of FSH. GnRH stimulation causes, indeed, excessive LH production [63] in those women. This condition may be determined by a higher frequency or amplitude of GnRH [60]. It is not yet clear whether alteration of the hypothalamo-pituitary axis in PCOS is primary or secondary to alterations in steroid hormones secretion. The role of FSH is to recruit ovarian follicles and stimulate their growth: 2–5 mm follicles are sensitive to FSH, whereas larger ones (6–8 mm) acquire aromatase activity and may increase oestradiol (E2) and inhibin B, reducing levels of FSH in late follicular stage. On the other hand, PCOS patients (having LH and FSH concentrations higher and lower than normal, respectively) accumulate antral follicles (2–8 mm) that differentiate early and undergo premature growth arrest [63]. Hypersecretion of LH in these women may promote early luteinisation of granulosa cells and contribute to early growth arrest of antral follicles (Fig. 3). LH may also activate premature meiotic processes that damage oocyte quality and contribute to the formation of embryonic aneuploidies [64].

Altered dopaminergic and opioid tone has also been found in these patients. However, administration of opioid antagonists or dopaminergic agonists in PCOS patients have little influence on LH pulsatility [63].

Among the excitatory elements of the reproductive axis, kisspeptins have recently emerged as essential upstream regulators of GnRH neurons, with indispensable roles in key aspects of reproduction, such as brain sex differentiation, puberty onset, gonadotropin secretion, ovulation, and the metabolic regulation of fertility [64–68]. Kisspeptins are a family of closely related peptides of different amino acid length (such as Kp-54 and Kp-10) that are encoded by the Kiss1 gene and operate through the G protein–coupled receptor Gpr54, also named kisspeptin receptor or Kiss1R [64, 65, 68]. Expression of the elements of the Kiss1 system in different ovarian compartments has been documented in human and rodent species [69, 70]. Ovarian expression of Kiss1 is under the positive control of gonadotropins [69]. On the other hand, local mediators also participate in the control of ovarian Kiss1 expression; inhibition of prostaglandin synthesis, which causes ovulatory dysfunction, evokes a marked suppression of ovarian Kiss1 mRNA levels during the periovulatory period. Moreover, inhibition of prostaglandin synthesis blocks the positive effect of gonadotropins on Kiss1 gene expression in the ovary [70].

Taken as a whole, these observations suggest a potential role of locally produced kisspeptins in the control of ovulation. Whether alterations of such local actions of kisspeptins might contribute to the ovulatory dysfunction seen in PCOS warrants specific investigation.

### Ovarian and extraovarian hyperandrogenism

Hyperandrogenemia is the most typical hormonal alteration of PCOS. Biochemically, hyperandrogenism is usually assessed by assay of total testosterone (TT), free testosterone (fT), sex hormone binding globulin (SHBG), androstenedione (A), 17-hydroxy progesterone (17-OHP) and dehydroepiandrosterone sulphate (DHEAS) in serum and by calculation of the free androgen index (FAI = (TT/SHBG)100). Women with PCOS often have higher than normal serum concentrations of all these androgens. Hyperandrogenism has a multifactorial origin attributed mostly to the ovaries with a substantial contribution from the adrenals and a minor contribution from fatty tissue.

**Fig. 4:**
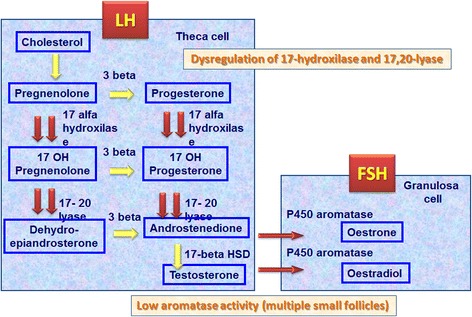
Relative inhibition of 17–20 lyase activity with respect to 17-hydroxylase has been found in women with PCOS. This leads to an increase in the 17OHP/A ratio and reduction of aromatase activity, the enzyme of granulosa cells that converts androgens into estrogens

Biosynthesis of androgens is mediated by microsomal P450c17 which catalyses 17–20 lyase activity. Alterations in P450c17 at transcriptional and post-transcriptional level have been implicated in the aetiology of PCOS [71]. These women show, indeed, relative inhibition of 17–20 lyase activity with respect to 17-hyroxylase, leading to an increased 17OHP/A ratio. Administration of GnRH or hCG in women with PCOS causes excessive production of 17OHP [68]. Low aromatase activity has also been demonstrated in women with PCOS. Aromatase is a granulosa cell enzyme that converts androgens into estrogens. It may be partly responsible for hyperandrogenism in this syndrome [70, 71] (Fig. 4).

Elevated levels of androgens may have a negative impact on follicular development, causing atresia, and on ovarian development, inhibiting meiotic maturation by decreasing oscillations of intracytoplasmic calcium levels [59].

### Hyperinsulinemia and insulin resistance

Insulin resistance is defined as a pathological condition in which a cell, tissue or organism requires above-normal quantities of insulin to respond normally. It causes increased insulin secretion by pancreatic β cells and compensatory hyperinsulinemia, while blood glucose remains normal. When the response of pancreatic cells decreases, the patient develops glucose intolerance or type II diabetes [5].

**Fig. 5:**
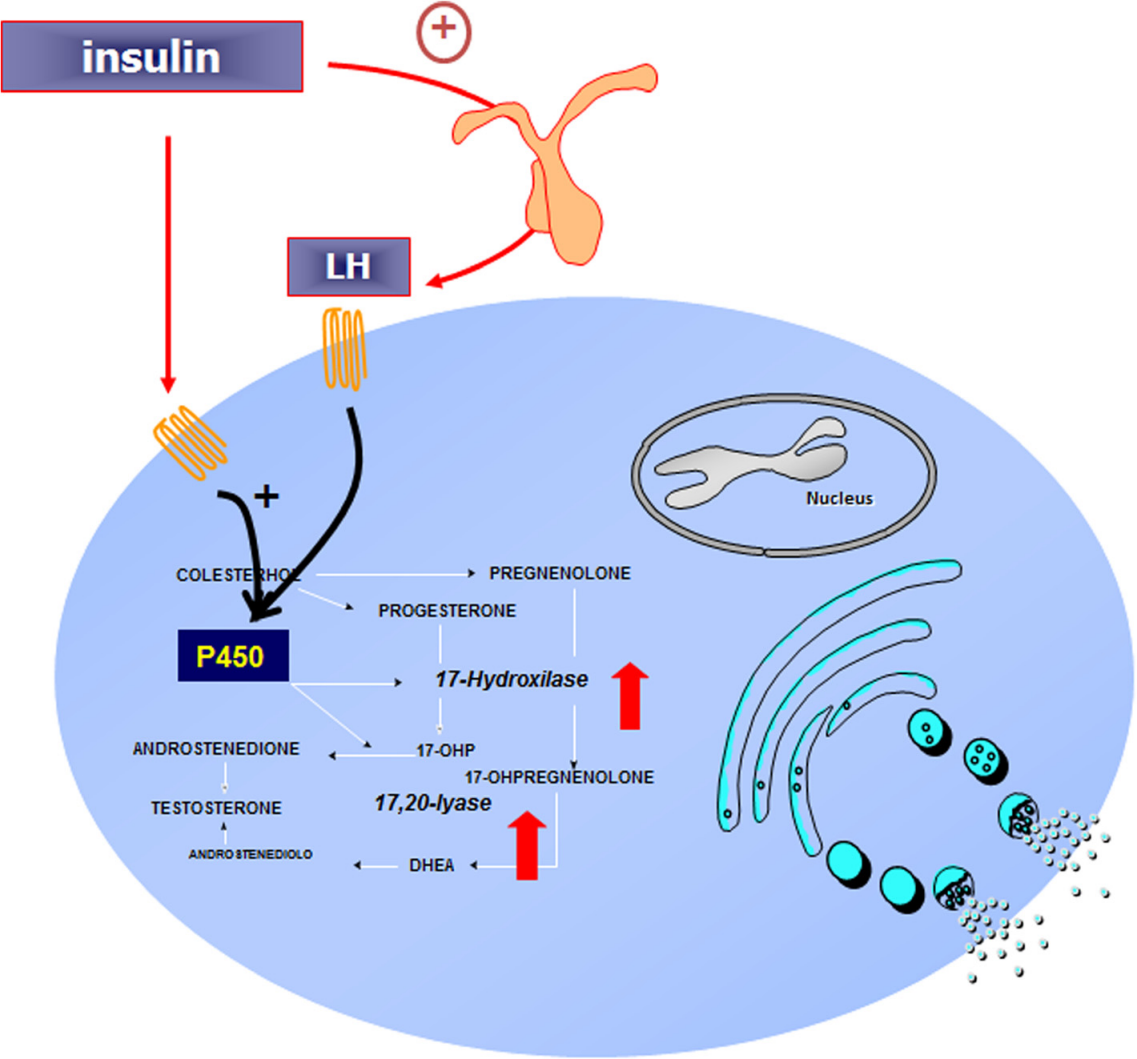
Hyperinsulinemia stimulates directly cytochrome p450 enzymes in the ovary or indirectly through action of LH or IGF-1, causing hyperandrogenism

Since many women with PCOS seem to have insulin resistance, compensatory hyperinsulinemia is thought to contribute to hyperandrogenism [72, 73] by direct stimulation of ovarian production of androgens and by inhibition of liver synthesis of SHGB that increases testosterone availability. Insulin also increases ACTH-mediated adrenal androgen production and accentuates LH-stimulated ovarian steroidogenesis [73] (Fig. 5).

About 60–70 % of women with PCOS are obese or overweight, and obesity is associated with insulin resistance. However, many studies have shown that insulin resistance is also present in many lean women with PCOS [5, 73]. The mechanisms leading to insulin resistance consist of a defect in insulin binding to its receptor or to changes in insulin signal transmission [5, 74]. However, the ovaries of these women maintain approximately a normal response to insulin. A partial elucidation of this mechanism is explained by the action of insulin on the ovary through the IGF-1 receptor. This binding occurs when insulin reaches high concentrations, as compensatory hyperinsulinemia. Moreover, the action of insulin on the ovary uses the inositol glycan system as a signal mediator, a different mechanism from the system activated by phosphorylation of the receptor at tyrosine level in other tissues [75]. An increase was observed in urinary clearance of inositol in some American and Greek women with PCOS. It reduces tissue availability of inositol. This mechanism could contribute to insulin resistance present in PCOS women [76]. Hyperinsulinemia directly stimulates ovarian steroidogenesis by acting on thecal and granulosa cells. *In vitro* studies have demonstrated that insulin stimulates thecal cell proliferation, increases secretion of androgens mediated by LH and increases cytochrome P450 expression of LH and IGF-1 receptor. Since the enzymes involved in ovarian steroidogenesis are similar to those of the adrenals, many studies have shown that insulin may act directly as stimulator adrenal steroidogenesis [5, 73]. The administration of metformin, an insulin-sensitising drug, significantly reduces production of 17OHP, T and A in response to ACTH in PCOS women [5].

*In vitro* data, obtained with cell culture models, indicate that co-incubation of insulin and FSH with bovine oocytes promotes up-regulation of LH receptors on granulosa cells of antral follicles. It contributes to arrest of follicular growth, inhibits of aromatase activity, and potentially triggers to ovarian hyperandrogenism. Looking at the molecular level, insulin binds to its receptor on granulosa and thecal cells and on oocytes where it may alter expression of certain genes involved in the meiotic process of the oocyte [59].

*In vitro* studies have demonstrated that insulin also has receptors at hypothalamus and pituitary levels, through which it stimulates the release of FSH and LH under basal conditions and after GnRH stimulation [5, 59].

Furthermore, insulin also influences hyperandrogenism by inhibiting liver synthesis of SHBG^2^ and IGFBP-1, which binds IGF-1 [5, 59] IGF-1 is a growth factor with endocrine action. It is mainly synthetized by the liver, but is also produced by other tissues, including the ovaries, where it has autocrine/paracrine functions. Many studies have shown a significant increase in the IGF-1/IGFBP-1 ratio in women with PCOS. An increased availability of IGF-1 in thecal cells can induce increased production of androgens. Moreover, IGF-1 stimulates oestrogen production by granulosa cells and synergically acts with FSH and LH in modulating expression of aromatase in granulosa cells. IGF-1, like insulin, also exerts an indirect control on ovarian steroidogenesis through the hypothalamus-pituitary axis. It induces, in fact, expression of GnRH and release of gonadotropins by the pituitary [5, 59]. Treatment with insulin-sensitising drugs increases IGFBP-1 levels, reduces the IGF-1/IGFBP-1 ratio and decreases IGF-1 availability in peripheral tissues [77].

### Intra and extra-ovarian factors

#### Epidermal growth factors (EGF)

Epidermal growth factor (EGF) plays an important role in regulation of cell growth, as well as in proliferation and differentiation through interaction with its receptor EGFR (ErbB1, ErbB2-4) [59, 71]. In the human ovary, EGF is present in follicular fluid (FF), where it regulates follicle development and oocyte maturation. In women with PCOS, FF EGF levels are higher than in normal ovulating women. In PCOS condition, EGF may inhibit granulosa cell oestrogen synthesis, which is translated into arrest of follicle growth [59, 71].

#### Insulin-like growth factors (IGF)

These growth factors are multifunctional polypeptides with insulin-like activity that play important regulatory functions for follicle and oocyte development [59, 71, 78]. Circulating IGFs are produced by the liver: IGF-1 is secreted by thecal cells, whereas IGF-2 is synthesised by granulosa cells and IGFBP (insulin-like growth factor binding protein) has been found in FF and is expressed by granulosa and thecal cells [78]. FF IGF-1 levels in PCOS women are elevated than in normal women, whereas IGFBP-1 are lower in PCOS patients, causing the arrest follicle growth [78].

#### Neurotrophin growth factor (NGF)

NGF is not only involved in development of the nervous system but also acts in the ovaries of humans and other mammals. It plays a fundamental role in folliculogenesis and oocyte maturation [59, 71, 78]. This factor promotes nuclear and cytoplasmic maturation of oocytes, and processes essential for the development of good quality oocytes and embryos. Elevated NGF concentrations in FF have been reported in women with PCOS [59, 71, 78]

#### Transforming growth factor-b (TGF-beta)

Members of the TGF-beta family play a role in follicle growth and oocyte development. They include anti-mullerian hormone, activin, follistatin, inhibins, bone morphogenetic protein (BMP)-9 and growth and differentiation factor (GDF)-9 [78, 79]. In different occasions, these growth factors may promote or block follicle growth and/or differentiation [74, 80].

#### Anti-Mullerian hormone (AMH)

AMH is a homodimeric glycoprotein that inhibits the development of Mullerian ducts in male embryos [59]. It is expressed by granulosa cells in the ovaries of women of reproductive age, where it controls the formation of primary follicles and follicle recruitment by FSH. Therefore playing an important role in folliculogenesis [74, 78, 79]. Women with PCOS have higher serum and FF concentrations of AMH compared to controls. This is closely correlated with greater development of antral follicles and arrest of follicular growth [79, 80]. High serum levels of AMH are directly correlated with an increase in testosterone and/or LH concentrations in women with PCOS, as well as with altered oocyte maturation and low embryo quality [64, 80]. Furthermore, elevated concentrations of AMH in FF of women with PCOS are correlated with a higher percentage of immature oocytes and lower fertilisation rates compared to women with endometriosis or pelvic adhesion syndrome [80].

#### Activin, follistatin and inhibin

Activin, follistatin (FS) and inhibin are polypeptides, which were originally isolated from ovarian FF. FS is a binding protein produced by ovarian granulosa cells; cellular growth and differentiation is regulated by autocrine/paracrine action. Over-expression of FS has been associated with arrest of follicle growth and reduced oocyte development. Activin is mostly secreted by smaller follicles. It promotes follicle development by increasing granulosa cell response to FSH stimulation; it decreases androgen synthesis and stimulating oocyte maturation. Besides inhibiting FSH production, inhibins are produced by the dominant follicle and stimulate thecal cells to produce androgens as substrate for estrogen formation [59, 74, 78, 79]. An increased FS/activin ratio and elevated concentrations of inhibin B have been found in PCOS [59, 74, 78, 79].

#### Vascular endothelial growth factor (VEGF)

VEGF is a homodimeric glycoprotein expressed in granulosa and thecal cells [78] and also present in FF [59]. It plays an important role in angiogenesis, follicular vascularisation and intra-follicular oxygenation. It has therefore an impact of follicle maturation, oocyte quality, fertilisation and embryo development [59, 74, 78, 79]. The alterations in FF concentrations of VEGF in patients with PCOS is indicative of oocyte immaturity [78]. The elevated concentrations are useful as an indicator of oocyte maturity, successful fertilisation and embryo development in women with PCOS [78].

#### Interleukins (IL)

Interleukins are a group of cytokines produced by leukocytes. In particular, IL-1, IL-2, IL-6, IL-8, IL-11 and IL-12 play different roles in the regulation of folliculogenesis, ovulation and corpus luteum function. Concentrations of IL-12 in FF differ from immature follicles and those in pre-ovulatory phase [78], and reduced FF concentrations of IL-12 and elevated FF concentrations of IL-13 in patients with PCOS are correlated with reduced oocyte maturation, fertilisation and pregnancy [59, 74, 78, 79].

#### Tumour necrosis factor α (TNF-α)

TNF-α is involved in the regulation of ovarian function, exerting an influence on proliferation, differentiation, follicle maturation, steroidogenesis and apoptosis. TNF-α is expressed by the oocyte, thecal cells, granulosa cells and the corpus luteum in the ovary. Alterations in TNF-α levels in FF are correlated to poor oocyte quality. Increased FF concentrations of TNF-α in women with PCOS are also significantly inverse correlated to FF concentrations of E2, which are indicative of poor oocyte and embryo quality [78].

#### Fas and Fas ligand (FasL)

Fas and FasL are membrane proteins belonging to a TNF subfamily, and they respectively have anti- and pro-apoptotic functions. Concentrations of Fas in FF are positively correlated with oocyte maturity [78]. In women with PCOS treated with metformin, a reduction in FF concentrations of FasL has been reported, with a consequent increase in implant and pregnancy percentages [59, 74, 78, 79].

#### Biomolecules related to carbohydrate metabolism

Proteomic studies show modulation of several proteins related to the carbohydrate metabolism. The abundance of many proteins such as aconitate hydratase, fructose bisphosphate aldolase A, malate dehydrogenase, isoenzymes M1/M2 of pyruvate kinase, and transaldolase has been found to be increased in ovarian tissue and in ovarian granulosa cells from PCOS patients. Instead, UDP-glucose 6-dehydrogenase protein was reduced in PCOS women.

Moreover, triosephosphate isomerise was shown to have increased gene expression in ovarian tissue from PCOS patients.

#### Biomolecules related to lipid metabolism

Women with PCOS frequently present an atherogenic serum lipid profile consisting of increased triglycerides, cholesterol, low-density lipoprotein cholesterol concentrations and reduced apolipoprotein A-I levels. Insulin resistance, androgen excess and obesity may all contribute to the abnormalities of lipid metabolism observed in women with PCOS.

Apolipoprotein A-1 (ApoA-I), the major structural protein component of HDL-cholesterol particles, has several pleiotropic biological functions: promotion of macrophage cholesterol efflux, stimulation of reverse lipid transport, inhibition of LDL oxidation, removal of toxic phospholipids and also many other anti-inflammatory properties. Proteomic techniques found a decreased abundance of ApoA-I in visceral adipose tissue and in whole ovarian tissue from women with PCOS [80–82]. Moreover, reduced ApoA-I abundance in granulosa cells from these women may influence the expression of steroidogenic enzymes and the production of the steroid hormone progesterone [83].

Apolipoprotein C-I (ApoC-I) inhibits lipoprotein metabolism in the liver. Several authors showed, thus, increased serum ApoC-I levels in women with PCOS compared to normal controls, especially in those presenting with insulin resistance [84].

Adipocyte plasma membrane-associated protein (APMAP) may represent a novel member of paraoxonases [85], which are known to be involved in antioxidant processes. Lower level of APMAP has been found in visceral adipose tissue in patients with PCOS [81] might contribute to the impairment in antioxidant defense characteristic of PCOS [86].

#### Biomolecules related to protein metabolism

Proteomic techniques indicated that ovarian tissue (in patients with PCOS) presents high levels of proteins involved in the metabolism of amino acids, in post-translational protein modification and in protein degradation [82].

Methionine adenosyltransferase II (MAT2B), an enzyme involved in the removal of homocystein, is decreased in the PCOS ovary [82]. Moreover, cathepsin D, an acid protease involved in intracellular protein breakdown implied in the pathogenesis of several diseases, such as, breast cancer [87, 88]; it is decreased in T lymphocytes from women with PCOS. The biological significance of this decrease is not clear [89].

### Other factors

#### Heat shock proteins (HSPs)

Heat shock proteins (HSPs) are a highly conserved family of molecules involved in protein folding. Many components of survival and apoptotic pathways are regulated by molecular chaperones such as heat shock proteins [90]. The decrease at the protein level of Hsp10, Hsp27 and Hsp60 in ovarian tissue and granulosa cells from patients with PCOS [79, 80] might contribute to apoptosis within the ovarian follicle. In accordance, Hsp60 is down-regulated in adipose tissue in PCOS gene expression studies [91].

#### Transferrin

Transferrin is the major iron transporter in the circulation and it is increased in PCOS women. High levels of trasferrin may not be related to inflammation but represent a compensatory mechanism against the limitation of iron availability for erythropoiesis characteristic of chronic disorders [92]. Similarly, the decrease in α2 macroglobulin observed in patients with PCOS might be related to the increased body iron stores, observed in these women [93, 94].

#### Homocysteine

Homocysteine (Hcy) is a homologue of the amino acid cysteine and may be converted into methionine or cysteine in the presence of B-complex vitamins. Many studies have shown that elevated Hcy levels in serum and FF are inversely proportional to oocyte and embryo quality [59, 74, 78, 79]. High FF and serum concentrations of Hcy may suppress E2 synthesis and therefore interfere with follicle growth and oocyte maturation in women with PCOS [59, 74, 78, 79].

#### Leptin

Leptin is a protein hormone that plays a key role in regulating energy supply and demand. High FF and serum concentrations of leptin are closely associated with a decrease in oocyte maturity and embryo quality in patients with PCOS. Certain studies have also demonstrated that high levels of leptin in women with PCOS play a role in PCOS pathogenesis, acting by inhibiting E2 production and interfering with follicle development and oocyte maturation. On the other hand, some authors have demonstrated that leptin is reduced in FF of women with PCOS and is therefore not a useful marker for evaluating oocyte quality [59, 74, 78, 79]. In-depth research is therefore needed to elucidate the role of leptin in the pathophysiology of PCOS.

#### Oxidative stress (OS)

Oxygen free radicals or reactive oxygen species (ROS) are involved in many physiological functions where they act as mediators in a variety of signal transduction pathways [59]. An excess of these substances can cause cellular damage. In women with PCOS, elevated levels of ROS in FF and reduced antioxidant capacity are closely associated with reduced oocyte maturation and low embryo quality [59, 74, 78, 79]. These molecules may reduce oocyte quality by altering the equilibrium of FFFs in the follicular microenvironment.

The decrease in mitochondrial O2 consumption and glutathione (GSH) levels, along with increased ROS production, explains the observed mitochondrial dysfunction in PCOS patients [95]. The mononuclear cells of women with PCOS are increased in this inflammatory state [96], which occurs more often in response to hyperglycemia and C-reactive protein (CRP). Physiological hyperglycemia generates increased levels of ROS from mononuclear cells, which then activate the release of TNF-α and increase inflammatory transcription factor NF-kappa B. As a result, the concentrations of TNF-α, a known mediator of insulin resistance, are further increased. The resulting OS creates an inflammatory environment that promotes insulin resistance and contributes to hyperandrogenism [97].

### Clinical manifestations

The typical clinical indications of PCOS are: anovulatory cycles, ultrasonographic evidence of polycystic ovaries and hirsutism. Many women are also overweight or obese and have an increased risk of developing metabolic syndromes in later life. During pregnancy, there is a higher chance of miscarriage, gestational diabetes and hypertension.

#### Anovulatory cycles

Anovulatory cycles often manifest with oligoamenorrhea, secondary amenorrhea or abnormal uterine bleeding. The term oligomenorrhea refers to cycles of more than 35 days, while secondary amenorrhea is an absence of menstruation for more than three months. Polymenorrhea condition, meaning more frequent cycles, generally with an interval of less than 24 days, may occur in a minority of cases. Since regular cycles do not exclude chronic anovulation, it is necessary to measure serum concentrations of progesterone in luteal phase of the cycle (days 20–24). If they are below 5 ng/mL, the cycle is probably anovulatory.

Menstrual irregularities often begin after menarche and decrease when the patient approaches menopause [98]. This correlated to a decline in androgen levels with advancing age in women with PCOS [98]. While evaluating the length of the menstrual cycle, it should be recalled that oligo-anovulation is quite common in adolescents, especially in the first year after menarche. Settling into regular cycles may be a slow process, which, in some cases, may take three years after the first period. This is why it is important to be cautious in diagnosing and treating PCOS in adolescents. The incidence of irregular cycles in adolescents with PCOS seems to vary significantly: about 43 % with oligomenorrhea, 21 % with primary or secondary amenorrhea, 21 % with regular menstrual cycles and 7 % with polymenorrhea [99]. 95 % of adult women with PCOS have amenorrhea [5, 99].

#### Ultrasonographic features of the ovaries

The Rotterdam guidelines of 2003 include ultrasonographic evidence of polycystic ovaries as a criterion for the diagnosis of PCOS. This finding is not exclusive because young healthy women may have ovaries with polycystic features. Polycystic ovaries may also be observed during pubertal development in patients with hypothalamic amenorrhea and hyperprolactinemia [100].

The diagnostic criteria of PCOS are based on the presence of 12 or more follicles of diameter 2–9 mm or an ovarian volume of more than 10 mL in follicular phase. Another feature is an increase in stromal tissue. These morphological changes in the ovary may be encountered in more than 80 % of women with a clinical diagnosis of PCOS [100].

#### Hirsutism

Hyperandrogenism may manifest with hirsutism, acne and alopecia. Hirsutism is the presence of terminal hair on the face and/or body in a masculine pattern. It is the most common symptom, found in about 60 % of women with PCOS and it widely varies according to the ethnicity. For this reason, the threshold of hirsutism should be set considering the patient ethnicity. The most widely used method to determine the degree of hirsutism is the Ferriman-Gallwey score [101] which gives a score of 0 in the absence of terminal hair in a given area of the body, and a score of 4 for extensive hair growth. Hair is scored in 9 different areas of the body, such as, chin, upper lip, periareolar and intermammary areas, upper and lower back, upper and lower abdomen, upper and lower limbs. The score from each area is summed to obtain a final score used for diagnosis. A score of 7 is indicative of hirsutism. It is defined as “slight” for scores of 7–9, “moderate” for 10–14 and “severe” for scores ≥15 [101].

Hirsutism may be simultaneously due to androgen production, increased circulating levels of free testosterone (Ft) in women with PCOS; together with an increased activity of androgens in the pilosebaceous units through action of 5-α reductase, an enzyme that transforms testosterone into the more active dihydrotestosterone.

#### Acne

Acne occurs in 12–14 % of women with PCOS and varies according to ethnicity: the highest reported incidence regards Indo-Asian women and lowest, Pacific islanders [102]. Acne consists of comedones, due to accumulation of sebum and epithelial cell debris, which is colonised by the bacterium *Propionibacterium acnes*. Inflammation of the comedones leads to the formation of papules, pustules and nodules. Androgens may exacerbate this process, increasing sebum production by pilosebaceous units. About 50 % of women with acne have no clinical or biochemical evidence of hyperandrogenism. Moreover, many hirsute women with PCOS do not have acne. These differences may be due to different peripheral sensitivity of androgen receptors [102].

#### Alopecia

Alopecia consists in progressive hair loss or thinning. The intensity varies from subject to subject. Androgenic alopecia is often accompanied by seborrhea and dandruff. Sensitivity of the pilosebaceous unit to androgens is highly variable and there is little correlation between clinical features and biochemical profiles of hyperandrogenism [102]. Hair loss in PCOS usually involves thinning at the vertex with maintenance of the frontal hairline.

### PCOS in adolescence and at menopause

It has been known for several years that PCOS patients have higher risk for a certain range of diseases compared to the general population. This risk exposes them to high morbidity and it is associated with high social impact, both economic and in healthcare. These pathologies include type II diabetes, metabolic syndrome, cardiovascular disease, endometrial carcinoma and many gestational complications. The clinical indicators of hyperandrogenism are another important aspect for adolescents with PCOS, considering of self-perception in this delicate period of life, when physical appearance is fundamental for self-acceptance and relationships with others. Hirsutism, acne and obesity cause psychological distress that may develop into personality disorders and depression. Early diagnosis and treatment of PCOS in adolescence is therefore fundamental because it can slow down or prevent the appearance of these pathologies in adulthood. Diagnosis of PCOS in adolescence is more problematical than in adulthood and, according to some authors, should be based on all three Rotterdam criteria [103]. Oligomenorrhea should have a history of at least two years since menarche and diagnosis of polycystic ovary by abdominal ultrasonography should only be based on increased ovarian diameter (>10 cm^3^). If diagnosis cannot be confirmed patients should be carefully monitored until adulthood, and if symptoms persist the diagnosis should be reassessed [103].

The clinical manifestations of PCOS in perimenopause period are not well known. There is histological evidence that women with PCOS have a larger cohort of primary follicles than healthy women of the same age, a greater number of antral follicles detectable by ultrasonography and higher serum concentrations of AMH [104]. These results suggest prolonged reproductive function and greater ovarian reserves. Women with PCOS also seem to achieve better menstrual regularity and probability of ovulation with age (despite lower pregnancy rates). A study of a prospective cohort of women during menopause shows that those with PCOS go into menopause an average of two years later than controls [104].

### Long-term sequelae

**Fig. 6:**
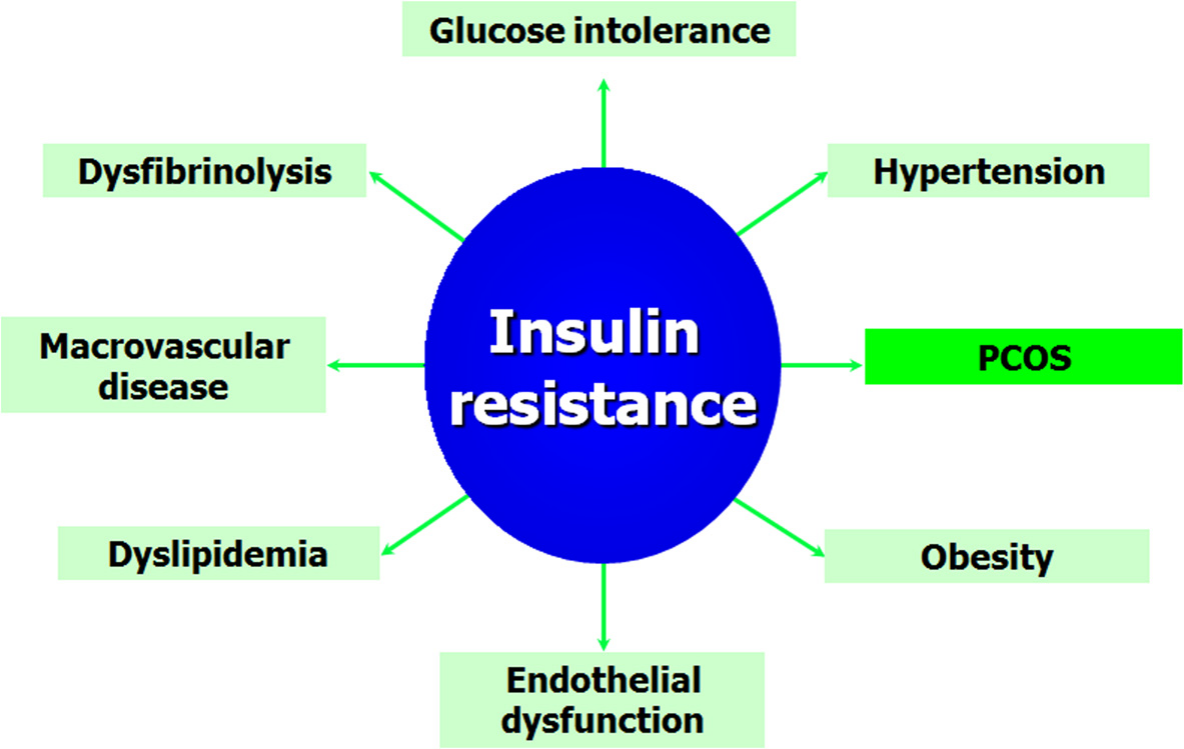
Insulin resistance is the link between PCOS and metabolic syndrome

Insulin resistance in young healthy women raises the problem of other risk factors such as impaired glucose tolerance (IGT), diabetes, hyperlipidemia, hypertension, abdominal obesity and risk of cardiovascular disease [5, 105]. Since PCOS patients tend to have abdominal fat deposition and insulin resistance, it has been suggested that they may also have other metabolic alterations typical of so-called metabolic syndrome. This syndrome is characterised by a series of symptoms, such as insulin resistance, obesity, hypertension and hyperlipidemia. Women with PCOS have elevated blood pressure, serum triglycerides, LDL, total cholesterol and lower HDL cholesterol than age-matched controls [5, 105]. Furthermore, PCOS patients have a seven times higher risk of myocardial infarction than controls of the same age (Fig. 6).

Insulin resistance is recognised as a major risk factor for type II diabetes [5, 105]. Factors such as obesity and family history of type II diabetes can increase the risk of diabetes in PCOS. About 30 % of obese women with PCOS have IGT. A retrospective study by Dahlgren et al. showed that prevalence of non-insulin-dependent diabetes mellitus (NIDDM) was 15 % in PCOS patients and 2 % in controls. Dunaif et al. [5, 105] suggested that up to 20 % of PCOS patients have IGT or NIDDM in the third decade.

It cannot be argued that PCOS patients with excess of androgens and anovulation are more vulnerable to metabolic dysfunction than other women. Women with PCOS and anovulation, but with normal levels androgens, and those with hyperandrogenism but regular cycles, usually have normal insulin sensitivity and presumably do not have the same risk of IGT or type II diabetes as those with the “classical phenotype” of the syndrome [106].

Besides, women with PCOS are also at higher risk for endometrial hyperplasia and carcinoma. This risk is, in fact, influenced by various factors, such as obesity, hyperandrogenism and infertility. All these factors can be present in women with PCOS. A recent prospective study of 56 PCOS patients showed a high prevalence of endometrial hyperplasia [5, 105]. An interesting recent review and meta-analysis confirms that women of all ages with PCOS have an increased risk of endometrial cancer but the risk of ovarian and breast cancer was not significantly increased [107]. For this reason, preventive measures on PCOS patients for endometrial carcinoma are suggested. These include recognition and treatment of relative hyperestrogenism by periodic administration of progesterone and/or ultrasonography and endometrial biopsy in cases of long periods of amenorrhea. In any case, at least four episodes of suspension bleeding per year (every 3 months) are recommended.

### PCOS and pregnancy: gestational complications

Women with PCOS show higher risk of gestational complications, such as miscarriage, gestational diabetes, hypertension and pre-eclampsia. These problems expose them to a higher risk of premature delivery and caesarean section [108]. Recent epigenetic theories suggest that during PCOS pregnancy the embryo is exposed to an excess androgens that disrupts functional reprogramming of foetal tissues [109, 110]. Maternal, placental or foetal hyperandrogenism can distressed epigenetic reprogramming of tissues, especially of genes regulating reproduction and metabolism. Which can contribute to diseases such as type II diabetes, hypertension, autism^31–32^ and PCOS. Epigenetic alterations of the androgen receptor gene on chromosome X have, indeed, been observed in women with PCOS. However, a recent study failed to find any significant differences in overall methylation of peripheral leukocyte DNA between women with PCOS and matched controls [109, 110], so aforementioned theory has not yet been confirmed.

**Fig. 7:**
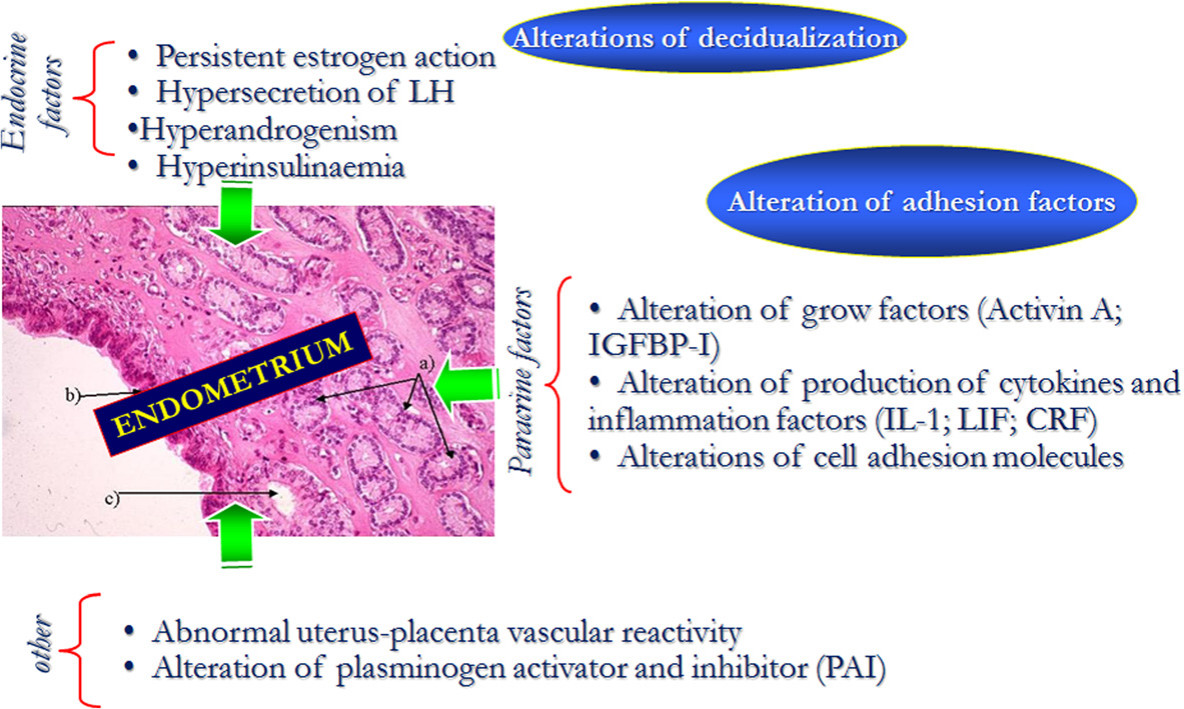
Factors involved in the etiopathogenesis of miscarriage in women with PCOS

Women with PCOS have a 30–50 % of risk of miscarriage, which is three times higher than normal women [111]. The mechanisms probably involved in the pathogenesis of miscarriage in these women are:

1. overexpression of androgen and steroid receptors and simultaneous reduced expression of molecules of implantation, such as α vs β 3 integrin and glycodelin [111];
2. hyperinsulinemia which inhibits endometrial and stromal differentiation *in vitro* (decidualisation) and locally down-regulates IGFBP-1 [111];
3. hypofibrinolysis mediated by high levels of plasminogen activator inhibitor (PAI) [111];
4. increased resistance of the uterine arteries blood flow leading to reduced sub-endometrial and endometrial vascularisation [111] (Fig. 7).

Moreover, women with PCOS have a higher incidence of gestational diabetes (20–30 %) and pre-eclampsia/pregnancy-induced hypertension (PE/PIH) (10–15 %) [108]. These alterations could be caused by obesity, alterations in glucose metabolism or in uterine vascularisation [112]. Obesity in pregnancy is, in fact, associated with various complications, such as miscarriage, pre-eclampsia, gestational diabetes, foetal macrosomia and caesarean section [112]. Fat tissue produces adipokines, including leptin, adiponectin, TNF-α, IL-6, resistin and visfatin, which could be involved in activation of insulin resistance in pregnancy. Adipokines can also produce an excessive local and systemic inflammatory reaction, which would play a key role in the pathophysiology of PE/PIH and the birth of SGA babies. It is also possible that placental macrophages contribute to inflammation within the placenta by secretion of pro-inflammatory cytokines such as IL-1, TNF-α and IL-6 in cytotrophoblast and syncytiotrophoblast cells [112].

Glucose intolerance and insulin resistance are elevated in women with PCOS even before pregnancy. Since pregnancy causes physiological insulin resistance through the action of placental hormones such as placental lactogen (hPL), placental growth hormone (hPGH) and progesterone, women with PCOS have even an higher risk of developing gestational diabetes. However, studies on the prevalence of gestational diabetes in women with PCOS show conflicting results that reflect the heterogeneity of the syndrome and the diversity of methods used to diagnose gestational diabetes [108].

Furthermore, alterations of uterine vascularisation reported in these women can determine reduced trophoblastic invasion leading to increased incidence of hypertension and delivery of SGA babies [112].

Maternal and placental hyperandrogenism may contribute to an increased risk of PE/PIH. In normal condition, the androgens synthesised by the placenta are rapidly converted to oestrogens by placental aromatase. Insulin inhibits placental aromatase and stimulates 3 βHDS activity. Expression of androgen receptors is significantly increased in the placentas of women with PE/PIH and this may induce vasoconstriction and thrombosis [108, 112]. Finally, all these complications expose pregnant women with PCOS to a greater risk of premature delivery and caesarean section [108].

### Outcome of newborns of mothers with PCOS

A meta-analysis aimed at evaluating neonatal complications in women with PCOS was recently conducted [108]. The newborns of PCOS patients had a significantly elevated risk of admission to the neonatal intensive care unit and a higher possibility of perinatal mortality [108]. Access to neonatal intensive care is partially linked to premature delivery, which causes hypoglycemia, jaundice and respiratory distress. Many women with PCOS also undergo ovulation induction and in vitro fertilisation and are therefore at chance for multiple pregnancies [113]. Multiple pregnancy is another major cause of increased perinatal morbidity. However, the studies aforementioned did not show any difference in multiple pregnancy between women with PCOS and healthy women [113].

Given the excessive rate of gestational diabetes in women with PCOS, an increased incidence of foetal macrosomia could be expected. However, newborns of women with PCOS show a significantly lower birth weight than controls, although the size of this difference (mean 40 g) is probably of limited clinical significance [108].

The foetus of PCOS mothers is exposed to greater glucose load, but placental distress can reduce the manifestation of macrosomia. The association of PCOS and PE/PIH suggests placental distress, especially in cases of preterm delivery [108]. The above-mentioned meta-analysis had some limitations regarding heterogeneity of PCOS patients enrolled in the different studies. BMI, medically assisted procreation and smoking during pregnancy were not always considered, all of which are factors that may affect obstetric and neonatal outcome [108].

## Conclusions

PCOS is not only a reproductive pathology but also a systemic condition and its etiopathogenesis is still not completely understood. Recently, the approach of clinical practice has been a progressive changed and improved towards prevention together with the standard treatments for diseases. Therapeutic tools are represented by hormonal contraceptives, antiandrogen drugs, metformin and inositols

In this context, PCOS is an excellent example of pathology in whih early diagnosis and treatment can prevent or delay its typical long-term sequelae.

In the past, therapy for PCOS has been centred on treatment of hirsutism and restoration of ovulation. However, it should be taken more into account the observation of hyperinsulinemia and insulin resistance, which are often implicated in the pathogenesis of the syndrome. Due to the fact that these alterations have major repercussions on health in the long period, the researchers should evaluate more appropriate strategies for control of the metabolic alterations of PCOS.

## Abbreviations

17-OHP, 17-hydroxy progesterone; A, androstenedione; AES, androgen excess society; AMH, anti-mullerian hormone; APMAP, Adipocyte plasma membrane-associated protein; ApoA-I, apolipoprotein A-I; BMI, body mass index; BMP, bone morphogenetic protein; CRP C, reactive protein; DHEAS, dehydroepiandrosterone sulphate; DM, diabetes mellitus; E2, oestradiol; EGF, epidermal growth factor; FAI, free androgen index; FasL, fas and fas ligand; FBN3, fibrillin-3; FF, follicular fluid; FFFs, follicular fluid factors; FS, follistatin; FSH, follicle stimulating hormone; Ft, free testosterone; GDF, growth and differentiation factor; GSH, glutathione; Hcy, homocysteine; HOMA, Homeostatic model assessment; Hpgh, placental growth hormone; hPL, placental lactogen; HSPs, heat shock proteins; IGF, insulin like growth factor; IGT, impaired glucose tolerance; IL, interleukin; IR, insulin resistance; KP, kisspeptin; LH, luteinising hormone; LHR, LH receptor; MAT2B, Methionine adenosyltransferase II; NGF, neurotrophin growth factor; NIDDM, non-insulin-dependent diabetes mellitus; NIH, National Institutes of Health; OS, oxidative stress; PCOS, policyctyc ovary syndrome; PE/PIH, pre-eclampsia/pregnancy-induced hypertension; ROS, reactive oxygen species; SGA, small for gestational age; SHBG, sex hormone-binding globulin; T, testosterone; TGF, transforming grow factor; TGF-beta, Trasforming growth factor beta; TNF-α, tumor necrosis factor α; TT, total testosterone; VEGF, vascular endothelial growth factor
